# NT-proBNP as predictor of major cardiac events after renal transplantation in patients with preserved left ventricular ejection fraction

**DOI:** 10.1186/s12882-023-03082-9

**Published:** 2023-02-11

**Authors:** Sebastian Schwab, Daniel Pörner, Carola-Ellen Kleine, Roxana Werberich, Louisa Werberich, Stephan Reinhard, Dominik Bös, Christian P. Strassburg, Sibylle von Vietinghoff, Philipp Lutz, Rainer P. Woitas

**Affiliations:** 1 Department of Internal Medicine I, Nephrology Section, University of Bonn, Bonn, Germany; 2 Kuratorium for Dialysis, KfH Renal Center, Bonn, Germany; 3 Diaverum Deutschland GmbH, Munich, Germany

**Keywords:** NT-proBNP, Renal transplantation, Major cardiovascular event

## Abstract

**Background:**

For the improvement of outcome after renal transplantation it is important to predict future risk of major adverse cardiac events as well as all-cause mortality. We aimed to determine the relationship of pre-transplant NT-proBNP with major adverse cardiac events and all-cause mortality after transplant in patients on the waiting-list with preserved left ventricular ejection fraction.

**Patients and methods:**

We included 176 patients with end-stage renal disease and preserved left ventricular ejection fraction who received a kidney transplant. MACE was defined as myocardial infarction (ST-segment elevation [STEMI] or non-ST-segment elevation [NSTEMI]), stroke or transient ischemic attack), coronary artery disease requiring intervention or bypass or death from cardiovascular causes.

**Results:**

MACE occurred in 28/176 patients. Patients with NT-proBNP levels above 4350 pg/ml had 1- and 5-year survival rates of 90.67% and 68.20%, whereas patients with NT-proBNP levels below 4350 pg/ml had 1- and 5-year survival rates of 100% and 90.48% (*p* < 0.01). 1- and 5-year MACE-free survival rates were calculated as 78.82% and 74.68% for patients with NT-proBNP > 4350 pg/ml and 93.33% and 91.21% for patients with NT-proBNP < 4350 pg/ml (*p* < 0.01).

**Conclusions:**

Pre-transplant NT-proBNP might identify renal transplant candidates at risk for MACE after transplant.

## Introduction

Renal transplantation is a treatment of end-stage kidney disease (ESKD), which largely improves quality of life and prolongs survival [1]. Recipients of a kidney transplant have a decreased incidence of cardiovascular events compared to patients remaining on the waiting list, whereas their cardiovascular risk is still higher than in the general population [2]. To improve outcome for renal transplant patients, estimation of future cardiovascular risk is therefore important. However, compared to the general population, risk assessment is inadequately delineated for patients with ESKD [3], especially for the period after kidney transplant in patients with preserved cardiac function. A widely available, simple biomarker to predict major adverse cardiac events (MACE) in renal transplant recipients would be very helpful to counsel patients and tailor individual preventive strategies.

Amino-terminal pro-B-type natriuretic peptide (NT-proBNP) is a marker of myocardial damage and fluid volume overload [4, 5]. Asymptomatic and euvolemic patients on maintenance dialysis often show NT-proBNP plasma levels greater than 20 times of the upper limit [3, 6]. NT-proBNP predicts cardiovascular morbidity and all-cause death both in the general population, and in chronic kidney disease [7–10]. However, the value of NT-proBNP as a reliable cardiac marker in patients with renal failure has been questioned [11–13], because NT-proBNP is at least partially cleared from the circulation by the kidneys [14, 15].

NT-proBNP was identified as a risk factor for mortality with an even stronger association in a cohort of renal transplant recipients than the general population [13]. In spite of its well-known association with major cardiac events (MACE) and mortality in ESKD patients, it is largely unknown if this association holds true for patients once they received kidney transplantation. Only few studies investigated this association. In contrast to the previous study [13], NT-proBNP was measured before transplantation in our cohort. Additionally, NT-proBNP at transplantation was found to be significantly higher in patients suffering from MACE very early after transplant, but this analysis comprised only few patients and analyzed MACE only within the first days after transplant [16]. Because cardiac insufficiency may be considered as a risk factor for MACE and mortality, we focused on patients without impaired left ventricular ejection fraction.

To further clarify the relationship between NT-proBNP and risk for MACE, we investigated pre transplantation NT-proBNP as potential predictor of MACE and mortality in patients with preserved left ventricular ejection fraction following renal transplantation.

## Patients and methods

### Study population

This retrospective study was performed at the University Hospital Bonn, Germany. Patients suffering from ESKD and wait-listed for kidney transplantation were included, if they were at least 17 years old at the time of kidney transplantation. NT-proBNP was measured during routine laboratory evaluation directly before kidney transplantation in every patient without a specific clinical event to trigger the analysis. Because the timepoint of analysis was determined by the availability of a donor organ, there was no specific interval to the dialysis sessions apart from the fact that specimens were not collected during dialysis. The period between NT-proBNP measurement and transplantation was no longer than 24 h.

For this study, only patients with pre-transplant measurement of NT-proBNP and with preserved left ventricular ejection fraction (assessed by echocardiography and defined as ≥ 55%) were included. Information on diastolic dysfunction was not collected.

We excluded patients without MACE who were lost to follow-up within the first six months after transplant. In total, 176 patients being transplanted between the year 2000 and 2015 met these inclusion criteria. In our cohort, only a minority of 4% of the patients (7/176) were transplanted pre-emptively while the vast majority of the patients (96%, 169/176) were on dialysis.

Baseline patient characteristics of this study cohort as well as pre-transplant biomarkers were extracted from medical files and records. Biomarkers were measured before transplant and evaluated retrospectively. The reference range for normal NT-proBNP values was < 125 pg / ml for patients under 75 years of age and < 450 pg / ml for patients older than 75 years.

Calculation of eGFR based on Cockroft-Gault equation while body surface area was estimated according to Du Bois.

### Outcome assessment

Post-renal transplant major cardiovascular event (MACE) and post-transplant all-cause mortality were the outcomes of interest. MACE was defined as (1) ST-segment elevation myocardial infarction (STEMI), (2) non-ST-segment elevation myocardial infarction (NSTEMI), (3) coronary artery disease needing intervention/bypass surgery, (4) stroke or transient ischemic attack, or (5) mortality due to a cardiovascular cause (defined as death due to coronary artery disease, sudden cardiac death or stroke).

Medical data were used to obtain information on MACE and the cause of death. Follow-up time was defined from the time of kidney transplant until death, loss to follow-up, or the last time their medical records were queried (1st May 2015).

### Statistical analysis

For categorical variables, baseline characteristics were presented as absolute number and frequency (percent). For normally distributed, continuous data, results are given as mean and standard deviation (SD). Non-normally distributed, continuous data are presented as median with interquartile range (IQR). Receiver operating characteristic (ROC) curve analysis was performed to assess the capacity of several continuous parameters to predict MACE. When suitable, cut-off values were derived from the point on the ROC curve with minimum distance to the upper-left corner.

Patients were divided into groups based on their pre-transplant serum NT-proBNP value and groups were compared using Chi-square, Fisher’s exact test or Student's t test, as appropriate.

Univariate Cox regression analysis was applied to identify potential determinants of MACE. The variables derived from univariate analysis were considered for multivariate analysis. Definite variable selection by multivariate Cox regression analysis was then based on backward elimination of variables with *p* > 0.05. Fulfilment of the assumption of proportional hazards was graphically assessed by analysis of the Kaplan–Meier curves and the corresponding log-minus-log plots. For the testing for proportional hazards, continuous data were stratified into two groups according to their means or the earlier identified cut-off values. Assumption of proportional hazards was considered as fulfilled when Kaplan–Meier curves did not cross and log-minus-log plots were parallel. All variates considered for and eventually included in the multivariate Cox regression model fulfilled the assumption of proportional hazards. Results of the Cox regression analyses were reported as hazard ratios (HR) with 95% confidence intervals and the corresponding *p*-value.

The Kaplan–Meier method was used to plot and analyse survival curves and to provide survival rates reported as Kaplan–Meier estimates including 95% confidence interval in square brackets. Comparison of the survival curves was performed using the log rank test.

A two-tailed *p*-value of 0.05 was considered statistically significant. SPSS version 28 was used for statistical analyses.

## Results

### Characteristics of the cohort

We included 176 patients in our study. Patients were followed-up over a median observation time of 38.23 months (IQR: 15.58 to 71.30 months) and mean age was 52.12 years (± 12.52 years). 56.25% of the cohort was male. In total, MACE occurred in 15.91% of included patients. All of these observed MACE were myocardial infarctions (3 ST-segment elevation myocardial infarctions, 22 non-ST-segment elevation infarctions and 3 not further specified myocardial infarctions). Patient characteristics as well as pre-existing conditions and laboratory values are given in Table 1.

**Table 1:** Patients characteristics

	no MACE (*n* = 148)	MACE (*n* = 28)	all (*n* = 176)	p
General parameters				
Age [years]	50.76 ± 12.69	59.32 ± 8.75	52.12 ± 12.52	< 0.01
Sex (f/m)	70/78	7/21	77/99	0.03
Cardiovascular risk factors				
Arterial hypertension	94.56% (139/147)	96.43% (27/28)	94.86% (166/175)	1
Diabetes	12.16% (18/148)	21.43% (6/28)	13.64% (24/176)	0.23
Hyperlipidemia	20.95% (31/148)	21.43% (6/28)	21.02% (37/176)	0.95
Active smoking or smoking history	27.70% (41/148)	39.29% (11/28)	29.55% (52/176)	0.22
Pre-existing conditions				
Coronary disease	10.14% (15/148)	67.86% (19/28)	19.32% (34/176)	< 0.01
Prior myocardial infarction	5.41% (8/148)	25% (7/28)	8.52% (15/176)	< 0.01
Atrial fibrillation	9.46% (14/148)	32.14% (9/28)	13.07% (23/176)	< 0.01
Prior stroke	5.41% (8/148)	7.14% (2/28)	5.68% (10/176)	0.66
Peripheral arterial disease	7.43% (11/148)	25% (7/28)	10.23% (18/176)	0.01
Laboratory parameters				
Creatinine [mg/dl]	8 ± 2.73	7.42 ± 2.05	7.91 ± 2.64	0.29
eGFR [ml/min/m^2^]	6.22 ± 2.39	6.46 ± 2.50	6.26 ± 2.40	0.64
NT-proBNP [pg/ml]	7878.28 ± 15,929.28	16,921.98 ± 26,485.87	9317.05 ± 18,231.27	0.09
Phosphate [mmol/l]	1.58 ± 0.50	1.44 ± 0.44	1.56 ± 0.49	0.17
Calcium [mmol/l]	2.34 ± 0.20	2.36 ± 0.20	2.35 ± 0.20	0.76
PTH [pg/ml]	228.66 ± 295.98	255.25 ± 226.60	232.25 ± 286.64	0.77
Cholesterol [mg/dl]	191.29 ± 48.18	210.96 ± 48.95	194.43 ± 48.69	0.06
HDL-Cholesterol [mg/dl]	45.97 ± 13.06	41.40 ± 11.15	45.33 ± 12.76	0.47
LDL-Cholesterol [mg/dl]	110 ± 45.33	122.80 ± 54.55	111.78 ± 46.06	0.57
Triglycerides [mg/dl]	208.34 ± 123.28	258.42 ± 224.53	216.19 ± 144.14	0.10
HbA1c [%]	5.36 ± 0.68	5.85 ± 0.48	5.43 ± 0.67	0.18
Uric acid [mg/dl]	4.29 ± 1.70	3.97 ± 1.49	4.24 ± 1.67	0.37
CRP [mg/l]	7.83 ± 19.19	7.64 ± 11.34	7.80 ± 18.17	0.96

**Fig. 1:**
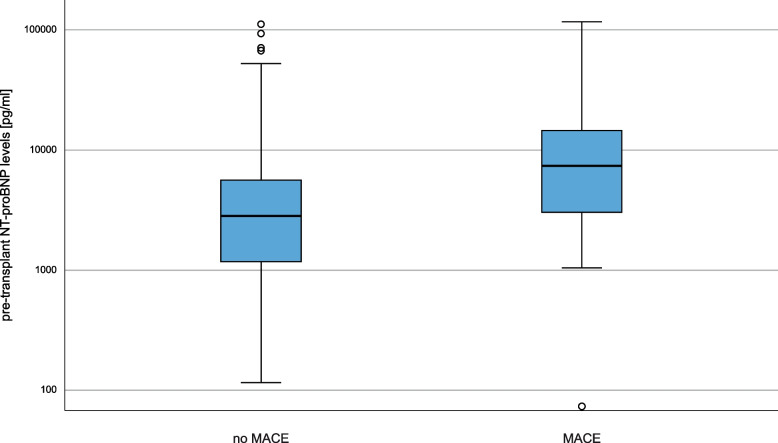
Distribution of pre-transplant NT-proBNP levels

Pre-existing conditions such as coronary artery disease, history of myocardial infarction, atrial fibrillation and peripheral arterial disease were more common in transplanted patients who developed MACE. NT-proBNP showed higher plasma levels in patients with future MACE, but shortly failed statistical significance (NT-proBNP without future MACE: 7878 ± 15,929 pg/ml versus NT-proBNP with future MACE: 16,922 ± 26,486 pg/ml; *p* = 0.09). Figure 1 visualizes the distribution of NT-proBNP levels for the cohort without vs. with future MACE. Based on eGFR and serum creatinine, kidney function was comparable between those with and without MACE (Table 1).

### NT-proBNP levels in patients with and without MACE

Median pre-transplant NT-proBNP level of patients who did not develop post-transplant MACE was 2829.50 pg/ml (IQR: 1173.88 to 5652.50 pg/ml) while patients who developed MACE post-transplant had a median NT-proBNP level of 7452 pg/ml (IQR: 2976.63 to 14,595.90 pg/ml). Note the logarithmic y-axis. Outliners are depicted as circles.

### Risk factors for MACE

Concerning laboratory markers no statistically significant differences between the groups with and without MACE could be observed (Table 1). Receiver operating characteristic curve determined the best cut-off value for NT-proBNP to predict MACE at 4350 pg/ml (area under the curve 0.69, sensitivity: 67.86%, specificity: 64.86%, positive predictive value: 26.76%, negative predictive value: 91.43%). Similarly, ROC curve analysis provided suitable cut-off values for recipient’s age at transplant (56.60 y, area under the curve 0.70, sensitivity: 67.86%, specificity: 66.22%, positive predictive value: 27.54%, negative predictive value: 91.59%) and cholesterol (200 mg/dl, area under the curve 0.62, sensitivity: 61.54%, specificity: 62.77%, positive predictive value: 23.88%, negative predictive value: 89.58%). Pretransplant triglyceride levels did not predict posttransplant MACE.

Univariate analysis identified age, male sex, coronary artery disease, prior myocardial infarction, atrial fibrillation and peripheral artery disease as risk factors of MACE (Table 2). Furthermore, pre-transplant serum NT-proBNP concentration > 4350 pg/ml was identified as risk factor for MACE in univariate analysis (Table 2). Interestingly, serum creatinine as well as creatinine-based eGFR did not associate with MACE.

**Table 2:** Risk factors of MACE

	Univariate analysis			Multivariate analysis		
	HR	95% CI	p	HR	95% CI	p
General parameters						
Age > 56,60 y	3.79	1.71; 8.40	< 0.01	2.47	1.05; 5.79	0.04
Male sex	2.46	1.05; 5.79	0.04	-	-	-
Cardiovascular risk factors						
Arterial hypertension	1.33	0.18; 9.83	0.78	-	-	-
Diabetes	1.87	0.76; 4.62	0.17	-	-	-
Hyperlipidemia	1.00	0.40; 2.46	0.99	-	-	-
Active smoking or smoking history	1.80	0.84; 3.87	0.13	-	-	-
Pre-existing conditions						
Coronary disease	12.89	5.77; 28.79	< 0.01	9.87	3.97; 24.51	< 0.01
Prior myocardial infarction	4.40	1.87; 10.39	< 0.01	2.82	1.04; 7.63	0.04
Atrial fibrillation	3.18	1.43; 7.06	< 0.01	-	-	-
Prior stroke	1.24	0.29; 5.24	0.77	-	-	-
Peripheral arterial disease	3.45	1.45; 8.16	< 0.01	-	-	-
Laboratory parameters						
Creatinine [mg/dl]	0.93	0.80; 1.08	0.36	-	-	-
eGFR [ml/min/m^2^]	1.02	0.88; 1.19	0.80	-	-	-
NT-proBNP > 4350 pg/ml	3.55	1.60; 7.88	< 0.01	3.04	1.29; 7.14	0.01
Phosphate [mmol/l]	0.59	0.25; 1.37	0.22	-	-	-
Calcium [mmol/l]	1.21	0.18; 8.16	0.85	-	-	-
PTH [pg/ml]	1.00	1; 1	0.76	-	-	-
Cholesterol > 200 mg/dl	2.59	1.17; 5.73	0.02	4.03	1.70; 9.56	< 0.01
HDL-Cholesterol [mg/dl]	0.98	0.90; 1.06	0.57	-	-	-
LDL-Cholesterol [mg/dl]	1.01	0.99; 1.02	0.48	-	-	-
Triglycerides [mg/dl]	1.00	1; 1	0.07	-	-	-
HbA1c [%]	2.24	0.63; 7.96	0.21	-	-	-
Uric acid [mg/dl]	0.89	0.69; 1.13	0.33	-	-	-
CRP [mg/l]	1.00	0.98; 1.02	1.00	-	-	-

In multivariate analysis, NT-proBNP above 4350 pg/ml was confirmed as independent risk factor for MACE (HR 3.04; *p* = 0.01). Beside NT-proBNP, also recipient’s age at transplant > 56.60 years (HR 2.47; *p* = 0.04), cholesterol above 200 mg/dl (HR 4.03; *p* < 0.01), coronary artery disease (HR 9.87; *p* < 0.01) and history of myocardial infarction (HR 2.82; *p* = 0.04) (Table 2) could be identified as risk factors for MACE.

To better characterize patients with high NT-proBNP levels, we compared patients with a pre-transplant NT-proBNP level > 4350 pg/ml to those below this threshold. Patients with NT-proBNP levels above the identified cut-off were of older age, suffered more often from atrial fibrillation and had lower triglyceride and uric acid level (Table 3).

**Table 3:** Comparison of cohorts with NT-proBNP above and below cut-off

	Pro-BNP ≤ 4350 pg/ml (*n* = 105)	Pro-BNP > 4350 pg/ml (*n* = 71)		all (*n* = 176)	p
General parameters					
Age [years]	50.24 ± 12.44	54.90 ± 12.20		52.12 ± 12.52	0.02
Sex (w/m)	42/63	35/36		77/99	0.22
Cardiovascular risk factors					
Arterial hypertension	48.02% (97/105)	49.64% (69/70)		48.68% (166/175)	0.32
Diabetes	11.76% (14/105)	12.35% (10/71)		12% (24/176)	0.89
Hyperlipidemia	17.32% (22/105)	17.44% (15/71)		17.37% (37/176)	0.98
Active smoking or smoking history	23.36% (32/105)	21.98% (20/71)		22.81% (52/176)	0.74
Pre-existing conditions					
Coronary disease	13.22% (16/105)	20.22% (18/71)		16.19% (34/176)	0.10
Prior myocardial infarction	7.08% (8/105)	8.97% (7/71)		7.85% (15/176)	0.60
Atrial fibrillation	5.41% (6/105)	19.32% (17/71)		11.56% (23/176)	< 0.01
Prior stroke	4.55% (5/105)	6.58% (5/71)		5.38% (10/176)	0.53
Peripheral arterial disease	7.08% (8/105)	12.35% (10/71)		9.28% (18/176)	0.16
Laboratory values					
Creatinine [mg/dl]	8.06 ± 2.74		7.69 ± 2.49	7.91 ± 2.64	0.37
eGFR [ml/min/m^2^]	6.31 ± 2.21		6.17 ± 2.67	6.26 ± 2.40	0.70
Phophate [mmol/l]	1.53 ± 0.47		1.60 ± 0.53	1.56 ± 0.49	0.37
Calcium [mmol/l]	2.35 ± 0.20		2.34 ± 0.19	2.35 ± 0.20	0.62
PTH [pg/ml]	186.97 ± 169.43		321.30 ± 423.70	232.25 ± 286.64	0.10
Cholesterol [mg/dl]	198.97 ± 40.86		187.76 ± 58.02	194.43 ± 48.69	0.15
HDL-Cholesterol [mg/dl]	44.83 ± 14.20		46.23 ± 10.21	45.33 ± 12.76	0.76
LDL-Cholesterol [mg/dl]	104.26 ± 34.83		125.08 ± 60.52	111.78 ± 46.06	0.27
Triglycerides [mg/dl]	236.79 ± 134.56		186.50 ± 153.09	216.19 ± 144.14	0.03
HbA1c [%]	5.36 ± 0.76		5.55 ± 0.51	5.43 ± 0.67	0.47
Uric acid [mg/dl]	4.50 ± 1.73		3.87 ± 1.52	4.24 ± 1.67	0.02
CRP [mg/l]	8.28 ± 21.75		7.08 ± 10.91	7.80 ± 18.17	0.67

### NT-proBNP and survival

**Fig. 2:**
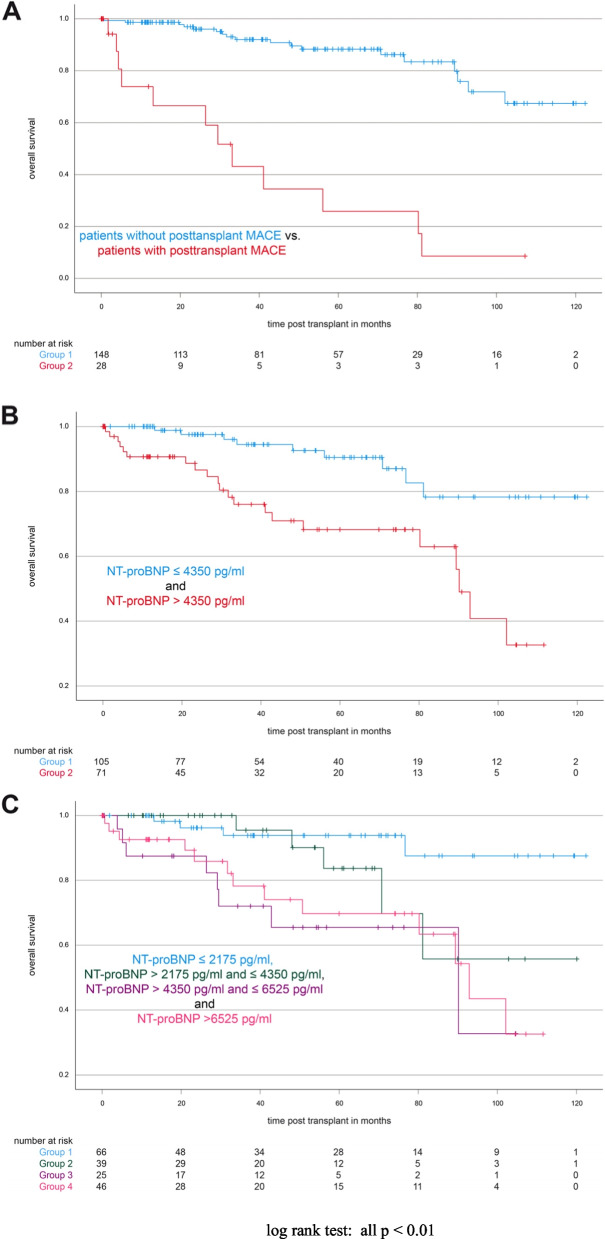
Overall survival. NT-proBNP and survival in a kidney transplantation cohort. **A** Kaplan–Meier curve of overall survival of patients with and without MACE; **B** survival of patients with NT-proBNP level lower or above 4350 pg/ml (*p* =  < 0.01); **C** survival of patients with given NT-proBNP values (*p* =  < 0.01)

Patients developing MACE over time showed a 1- and 5-year survival rate of 73.95% [62.70%; 85.20%] and 25.88% [13.40%; 38.37%], respectively. 1- and 5-year survival rates for patients without MACE were 98.65% [97.70%; 99.60%] and 88.25% [84.98%; 91.51%] (log rank test: *p* < 0.01, see Fig. 2A).

To analyse if our identified NT-proBNP cut-off discriminates between patients at higher risk of death, we plotted Kaplan–Meier curves for patients with NT-proBNP below and above our threshold of 4350 pg /ml. Patients with NT-proBNP levels above 4350 pg/ml had 1- and 5-year survival rates of 90.67% [87.04%; 94.30%] and 68.20% [61,38%; 75.03%], whereas patients with NT-proBNP levels below 4350 pg/ml had 1- and 5-year survival rates of 100% and 90.48% [86.68%; 94.27%] (log rank test: *p* < 0.01, see Fig. 2B).

To further adress this finding, we stratified the patients according to their NT-proBNP levels in four groups based on the earlier identified cut-off value. NT-proBNP levels inversely correlated with survival (pooled log rank test: p < 0.01, see Fig. 2C).

### NT-proBNP and MACE-free survival

**Fig. 3:**
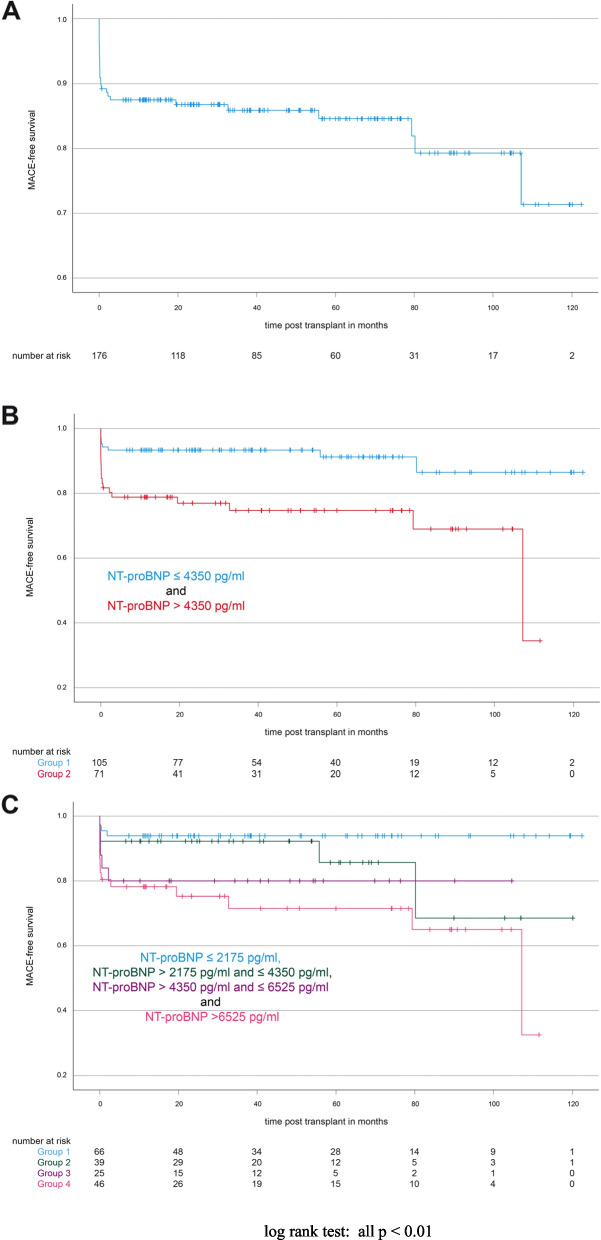
MACE-free survival. NT-proBNP and incidence of MACE in a kidney transplantation cohort. **A** Kaplan–Meier curve of MACE-free survival in our cohort; **B** MACE-free survival of patients with NT-proBNP level lower or above 4350 pg/ml (*p* =  < 0.01); **C** MACE-free survival of patients with given NT-proBNP values (*p* =  < 0.01)

Figure 3 illustrates MACE-free survival of all patients irrespective of their pre-transplant serum NT-proBNP levels. To analyze whether or not serum NT-proBNP levels correlate with MACE-free survival, we again plotted Kaplan–Meier curves for patients with NT-proBNP below and above the identified cut-off value of 4350 pg/ml (Fig. 3B). 1- and 5-year MACE-free survival rates were calculated as 93.33% [90.90%; 95.77%] and 91.21% [88.04%; 94.38%] for patients with NT-proBNP < 4350 pg/ml and 78.82% [73.97%; 83.68%] and 74.68% [69.26%; 80.10%] for patients with NT-proBNP > 4350 pg/ml (log rank test for the Kaplan–Meier curves: *p* < 0.01). Again, we further stratified the patients according to their NT-proBNP levels in four groups (Fig. 3C). The corresponding Kaplan–Meier curves differed significantly (pooled log rank test: *p* = 0.01) and MACE-free survival inversely correlated with NT-proBNP levels.

1- and 5-year MACE-free survival rates were the highest in patients with NT-proBNP ≤ 2175 pg/ml (both 93.94% [91%; 96,88%]) and the lowest in patients with NT-proBNP ≥ 6525 pg/ml (78.20% [72.10%; 84.30%] and 71.54% [64.34%; 78.74%], respectively). Patients with NT-proBNP between 2175 pg/ml and 4350 pg/ml (1- and 5-year MACE-free survival rates: 92.31% [88.04%; 96.57%] and 85.71% [78.23%; 93.20%], respectively) and between 4350 and 6525 pg/ml (1- and 5-year MACE-free survival rates: both 80% [72%; 88%]) showed intermediate MACE-free survival rates compared to the groups of low and high NT-proBNP (Fig. 3C).

## Discussion

Pre transplant systolic dysfunction is associated with mortality after transplantation [17]. But even within the group of patients with preserved left ventricular ejection fraction, significant differences concerning the risk for future cardiovascular events exist. Therefore, biomarkers at the time of transplantation may identify patients at greater risk for cardiovascular complications and for death. In a recent study, NT-proBNP, renal function and the need for anti-hypertensive medication have been identified as stronger risk factors for mortality in a cohort of renal transplant recipients in comparison to the general population [13], but all laboratory parameters have been measured at various time points after transplantation. In our study aiming at the identification of a predictive factor, NT-proBNP was measured shortly before transplantation.

In our cohort of 176 adult renal transplant recipients with preserved left ventricular ejection fraction we demonstrated a significant association between pre-transplant NT-proBNP and post-transplant MACE and confirmed NT-proBNP as independent risk factor for MACE and survival. Our findings are in line with previous studies, which showed that greater elevations of NT-proBNP were associated with higher risk for cardiovascular mortality [3]. Since NT-proBNP is released upon ventricle stretch as well as myocardial damage and is at least partially cleared by the kidneys, it is usually measured above tresholds in ESKD. This raises two main questions. Firstly, if a new biomarker stratification is needed especially for wait-listed patients, because the common thresholds do not apply in these patients. Secondly, how to interprete pre-transplant NT-proBNP results in patients after renal transplantation.

Here we could identifiy a pre-transplant NT-proBNP cut-off of 4350 pg/ml to predict MACE-free survival as well as overall survival in patients after renal transplantation. Therefore, NT-proBNP might help to identify renal transplant recipients at greater risk for cardiovascular mortality.

We further suggest that NT-proBNP as a widely available biomarker can be used for identification of patients at cardiovascular risk pre-transplantation regardless of their medical history or preexisting coronary artery disease. In our cohort, one third of patients with a typical history did not develop MACE after kidney transplant, so that NT-proBNP measurement may help to stratify patients better. However, our identified cut-off value has to be validated in a larger cohort and a prospective study design.

The relatively low overall NT-proBNP value are most likely due to the inclusion of patients with preserved left ventricular ejection fraction. For patients with heart failure and reduced ejection fraction (HfrEF) NT-proBNP values are much higher, so that NT-proBNP is an established diagnostic and prognostic marker of congestive heart failure and left ventricular systolic dysfunction [18]. Therefore, it is essential for the use of NT-proBNP as a prognostic marker to define appropriate subgroups according to the heart function. Within our study, we do not have information on diastolic dysfunction or signs of cardiac hypertrophy leading to increased NT-proBNP levels. However, since BNP was not closely related to diastolic dysfunction in a population based study, the presence of diastolic dysfunction would not be expected to bias our results considerably [19]. Up to date, there is a lack of published data regarding the relationship between NT-proBNP concentrations and diastolic dysfunction in ESKD patients [20]. In conclusion, we could demonstrate that especially in our cohort of relatively healthy patients with preserved left ventricular ejection fraction, pre-transplant NT-proBNP is a valuable tool to identify transplant recipients at high cardiovascular risk.

We found that extreme NT-proBNP values classified patients better than intermediate values. In studies comprising even larger number of patients, these less marked differences may become more prominent.

Although we cannot provide longitudinal data, analysis of NT-proBNP trajectory after renal transplantation might be of great interest. As demonstrated previously, an association of change in NT-proBNP at various time points and mortality is well established [21–23]. This effect was not limited to cohorts of dialysis patients. Also in a cohort of patients with CKD 3–4, patients with th greatest change in NT-proBNP had the poorest survival [24].

In our cohort, serum creatinine as well as creatinine-based eGFR did not show any difference between the groups of patients with and without MACE. Creatinine-based eGFR is of limited accuracy in describing residual renal function in ESKD patients. Interestingly, it has been shown that NT-proBNP had more influence on the difference in mortality between renal transplant recipients and the general population than creatinine clearance [13].

This is in line with a most recent published study of our group [25]. Pre-transplant residual renal function assessed by serum creatinine and serum cystatin C values were not significantly associated with MACE, whereas another biomarker of kidney function Beta-Trace protein (BTP) was. BTP has been demonstrated to be tightly associated with cardiovascular risk [26], which might explain these findings. In the cohort analyzed here, BTP was not associated with cardiovascular risk (data not shown), most likely because only patients with preserved left ventricular ejection fraction were included.

Also limitations of our study should be mentioned. Due to the observational design of our study, we cannot draw conclusions about the causal associations between pre-transplant NT-proBNP levels and post-transplant MACE as well as survival. Blood was taken immediately before transplantation, however we cannot rule out moderate differences with regard to excess volume depending on the last dialysis treatment, which might have taken place from 0 up to 72 h before transplantation. All patients were treated to the standard of care, so that an imbalance in cardioprotective medication between the NT-proBNP groups, which was not related to the previous diagnosis of cardiovascular disease, is unlikely. However, no prescribing information is available.

In conclusion, pre-transplant serum NT-proBNP as a widely available biomarker is associated with post-transplant MACE as well as survival. Interpretation of pre transplant NT-proBNP might help to identify patients at high risk for an undesired outcome.

## Data Availability

The datasets generated during and/or analyzed during the current study are available from the corresponding author on reasonable request.
